# Assessment of Serum Magnesium Fractions in Workers Exposed to Pb from Pb-Battery Plant

**Published:** 2018-11-18

**Authors:** Ravibabu Kalahasthi, Barman Tapu

**Affiliations:** ^1^ Regional Occupational Health Centre (Southern) ICMR complex, Poojanahalli Road, Kannamangala Post, Devanahalli, Bangalore, Karnataka, India

**Keywords:** Serum total magnesium, Ionized magnesium, Corrected magnesium, Pb-exposure, Pb- battery plant, lifestyle factors

## Abstract

**Background:** Most of studies assessed the effect of Pb-exposure on serum total magnesium (tMg). The hypomagnesium condition depended on protein concentration in the sample and influence of lifestyle factors. This study assessed the effect of Pb- exposure on serum tMg, corrected Mg (cMg), ionized Mg (iMg), percentage of iMg from tMg, and percentage of iMg from cMg with contemplation of lifestyle factors.

**Study design:** Case control study.

**Methods:** The serum magnesium fractions were assessed in 176 male Pb-exposed workers in the year 2015 at Tamil nadu in India and 80 control subjects with no occupational exposure of Pb. The serum tMg and albumin concentrations were estimated using diagnostic kit methods. Blood lead levels (BLLs) were estimated using atomic absorbtion spectrophotometer method. The fraction of cMg and iMg were calculated from serum tMg and albumin concentration among individual subjects.

**Results:** The BLLs was significantly (*P*<0.001) increased in the study group as compared to control. Serum tMg, cMg, iMg, % of iMg from tMg and % of iMg from cMg concentrations were not significantly decreased in the study group as compared to control. Pb-exposure was significantly associated with abnormal frequency distribution of serum iMg (*P*=0.048) and % of iMg from tMg (*P*=0.016). Smoking habit was significantly associated with cMg (*P*=0.039) and % of iMg from cMg concentration (*P*=0.018). The alcohol consumption was significantly (*P*=0.049) associated with cMg.

**Conclusion:** The Pb-exposure and lifestyle factor such as smoking and alcohol consumption were associated with alteration of serum magnesium fractions.

## Introduction

Magnesium (Mg) is second most abundant intracellular cation in the body. The highest percentage of body Mg is found in bones followed by muscle and soft tissue^1^. It is a divalent cation and plays critical role in calcium and potassium transport, cell signaling, energy metabolism, gene stability, DNA repair, and replication^2^. Mg exists in blood in three forms such as ionized fraction (iMg), which comprises about 55% of serum tMg, the Mg bound to protein, particularly to albumin about 20% and Mg complexed to anions about 25%. These three fractions are in equilibrium with each other^3^. The serum fractions of tMg and iMg were closely related in hypermagnesaemia and poorly related in hypomagnesaemia, which depends on protein concentration^4^. The measurement of serum tMg overestimates the incidence of hypomagnesaemia when hypoalbuminemia is present^5^. Chronic alcoholics found reduced levels of serum tMg and iMg concentration than in the control^6^. The deficiency of serum iMg was associated with acute migraines^7^. Serum iMg, quotient Mg (iMg/tMg) and bound-Mg concentration were noted reduced levels in acute myocardial infarction patients and serum tMg was not altered^8^. Significantly decreased levels of iMg and bound-Mg (tMg-iMg) were reported in hyperthyroid patients^9^.

About 3% increase of serum tMg concentration was reported in short-term occupational Pb-exposure^10^. The elevation of tMg caused by the increased release of Mg from the tissue due to its displacement from binding sites by Pb ions. Significantly decreased serum tMg and thiamine concentration was presented in Nigerian population exposed to Pb through their occupation and the low BLLs can enhance Pb absorption and also potentiate Pb-neurotoxicity in the presence of decreased serum tMg^11^. Significantly decreased of erythrocyte Mg was reported in workers, who had BLLs >20 µg/dL^12^. Moreover, decreased serum tMg was reported in occupational Pb- exposure^13^. In Pb- poisoning cases a significant drop of plasma Mg and without alteration of erythrocyte and urinary-Mg was reported^14^. Moreover, severe hypomagnesaemia was reported with persistent urinary loss of Mg in Pb-poisoning case^15^. Rats under subchronic Pb- intoxication were noted significantly reduced Mg in hard tissue and this reduction was due to competitive antagonism between Pb and Ca and Mg^16^. Rat intoxicated with ethanol plus Pb presented significantly decreased serum tMg, so it was concluded that the Pb-exposed human subject abusing alcohol might be vulnerable to accumulation of Pb in organs of the body and deficiency of bio-elements was associated with health injury^17^.

In some studies, hypomagnesium was also associated with oxidative stress, pro-inflammatory state, endothelial dysfunction, platelet aggregation, insulin resistance^18^, obesity^19^, acute exacerbation of COPD^20^, cardiovascular disease^21^, diabetes^22^, coronary artery disease^23^, lipid profiles^24^ and smoking^25^. Mg deficiency was associated with reduction of serum sphingomyelin with elevations of lipid profiles (Cholesterol, LDL-C,VLDL-C and triglycerides) and oxidative stress, characterized by reduction in glutathione (GSH) and activation of e-NOS and n-NOS^26,27^.

Studies on short-term Pb-exposure showed an increased serum t-Mg. The chronic and Pb-poisoning case showed decreased concentration of serum tMg. The hypomagnesium condition was dependent on protein concentration^4^. Incidence of hypomagnesium overestimates the serum tMg measurement when hypoalbuminemia is present^5^. Serum tMg measurement does not reflect the biologically active Mg^2+^ fraction. Serum iMg measurement provides better discrimination in normal and abnormal patients^28^. Decreased level of serum tMg was also associated with lifestyle factors such as obesity, smoking , alcohol consumption, hypertension and diabetes.

The present study has chosen the serum magnesium fractions such as tMg, iMg, cMg, % of iMg from tMg and % of iMg from cMg to assess the effect of Pb-exposure and lifestyle factors among workers from Pb- battery plant.

## Methods

This case-control study, we enrolled 176 male Pb-battery manufacturing workers in the year 2015 at Tamil nadu in India and considered them as study group and 80 healthy subjects with no occupational exposure to Pb considered them as a control group. Serum Mg fractions were compared between study and controls.

The institutional Ethical Committee (IEC) approved the study with letter no.142/6/dated 3-12-2014. Subjects were informed about the study and consent was obtained before their participation in the study.

Using the mean difference of serum tMg reported in occupational Pb-exposure and controls the sample size was calculated^13^. Total sample size obtained for this study was198 with 119 study and 79 control samples. Sample size was calculated using openEpi info, version 3 with input data of confidence interval (CI) 85%, power 80%, allocation ratio 1.5 and difference between means is 0.1. The subjects with risk of cardiovascular disease, thyroid dysfunction, and diabetes were excluded.

### Blood lead

The blood lead levels (BLLs) used as an indicator of Pb-exposure. The BLLs was measured using the method of Barman et al^29^. In this method, two ml of whole blood sample was digested by a microwave digestion system (ETHOS-D, Italy) with 2 mL of nitric acid (HNO_3_) and 0.2 mL of hydrogen peroxide (H_2_O_2_). The digested samples were made up to 5 mL using triple distilled water and centrifuged. The BLL was measured by an atomic absorption spectrophotometer (GBC-Avanta, Australia). Twenty µg/dL of the standard solution were prepared from the lead standard solution and added to the lowest concentration of the sample. The analysis found 100% recovery with % of relative standard deviation at <0.5 for three replicates. The frequency distribution of BLLs among study and control groups were done by using OSHA standard^30^.

### Body mass index and Blood pressure

BMI was calculated by using subjective weight (kg) and height (m) and expressed as Kg/m^2^. Thefrequency distribution of BMI and blood pressure (SBP & DBP) among study and control group did by using WHO classification^31^ and JNC 7^th^ report^32^ respectively.

### Serum magnesium

Serum total magnesium was determined using colorimetric and end point method. The diagnostic kit was manufactured by Linear Chemical SL, Joaquim Costa 18.2 planta, 08390 Montag, Barcelona, Spain. In this method, the specific binding of Calmagite, a metallochromic indicator and magnesium at alkaline pH with the resulting shift in the absorption wavelength at 520 nm. Intensity of the color formed is proportional to the concentration of Mg in the sample. Magnesium concentration in the samples was expressed as mg/dL. The detection limit of method is 0.01 mg/dL and linearity is up to 10 mg/dL. Corrected magnesium (cMg) and ionized magnesium (iMg) was calculated using serum tMg and albumin concentration with formulas^.^

(1) cMg (mg/dL) =Total magnesium -0.707 X (albumin-3.4)

(2) iMg (mg/dL)=[0.9+(0.55X total magnesium)-(0.3X albumin)]

### Statistical analysis

All the data were analyzed SPSS version 20. The data was presented in mean and standard deviation and proportion. Independent *t*-test was used to find out the differences in age and serum Mg fractions between study and controls. Chi-square test was applied to show differences in BMI, SBP, DBP, BLLs, smoking, alcohol consumption and serum Mg fractions between study and controls. Spearman’s correlation coefficient test was used to find out the association between BLLs and serum Mg fraction in study and control. The probability of less than 0.05 is considered as significant.

### Serum albumin

The serum albumin concentration was measured by using Prietest clinical chemistry reagents^33^. This diagnostic kit was manufactured by Robnik (India) private limited, industrial area, Mahape, Navi Mumbai, India. In this approach, albumin in a buffered solution reacts with the anionic Bromocresol green dye and gives a green color measured at 628 nm. The intensity of green color was directly proportional to concentration of albumin present in the sample. The results were expressed as g/dL of sample.

### Serum magnesium fraction

Percentage of iMg from tMg and percentage of iMg from cMg were calculated using the values off tMg, iMg and cMg.

## Results

The characteristics of study and control groups are presented in Table 1.Variables of BMI, blood pressure (SBP & DBP), smoking and alcohol consumption of study group was suitable matched with control. BLLs in the study group were significantly (*P*<0.001) increased as compared to control.

**Table1:** Characteristic of the study and control groups

**Variables**	**Study group(n=176)**	**Control group(n=80)**	***P*** ** value**
Body mass index(Kg/m^2^)			1.000
Normal (18.5-24.9)	77	35	
Overweight/Obese(≥25)	99	45	
Systolic blood pressure (mmHg)			0.463
<140	150	65	
>140	26	15	
Diastolic blood pressure (mmHg)			0.823
<90	158	71	
>90	18	09	
Blood lead levels (µg/dL)			0.001
<40	127	80	
>40	49	00	
Smoking			0.747
Yes	038	19	
No	138	61	
Alcohol consumption			0.500
Yes	95	39	
No	81	41	

The levels of serum magnesium fraction such as serum tMg, cMg, iMg, % of iMg from tMg and % of iMg from cMg are reported in Table 2. The levels of serum Mg fractions (serum tMg, cMg, iMg, % of iMg from tMg and % of iMg from cMg) were not significantly decreased in the study group as compared to controls.

**Table 2:** Serum magnesium fractions of study and control groups

**Variables**	**Study group(n=176)**		**Control group(n=80)**		***P*** ** value**
	**Mean**	**SD**	**Mean**	**SD**	
Serum total magnesium(mg/dL)	2.33	0.50	2.34	0.40	0.800
Serum ionized magnesium(mg/dL)	0.94	0.35	0.96	0.26	0.743
Serum corrected magnesium(mg/dL)	1.10	0.87	1.20	0.87	0.500
% of Ionized Mg from total Mg	39.42	10.55	40.47	5.60	0.301
% of ionized Mg from corrected Mg	48.87	9.88	49.86	2.10	0.377

The normal and abnormal frequency distribution of serum Mg fraction was done using 5th percentile of control group. The abnormal frequency of serum iMg (*P*=0.048 is equal to one-tailed) and % of iMg from tMg (*P*=0.016 is equal to two-tailed) levels were significantly decreased in the study group as compared to controls (Table 3).

**Table 3:** Normal and abnormal frequencies distribution of serum magnesium fraction of study and control group

**Cut-off value**	**Study (n=176)**	**Control (n=80)**	***P *** **value**
Serum total magnesium(mg/dL)			0.378
<1.5	08	06	
≥1.5	168	74	
Serum ionized magnesium(mg/dL)			0.048
<0.5	22	4	
≥0.5	154	76	
Serum corrected magnesium (mg/dL)			0.099
<0.16	19	6	
≥0.16	157	74	
% of Ionized Mg from total Mg			0.016
<30	25	03	
≥30	151	77	
% of Ionized Mg from corrected Mg			0.230
<47	18	06	
≥47	158	74	

Smoking habit significantly decreased the levels of cMg and % of iMg from cMg. Alcohol consumption was significantly decreased cMg.The other lifestyle factors such as BMI, SBP,and DBP among these subjects didnot influence the serum magnesium fraction (Table 4).

**Table4:** Serum magnesium fraction in Pb-exposure and lifestyle factors

**Variables**	**n**	**tMg** **(mg/dL)**		**iMg** **(mg/dL)**		**cMg** **(mg/dL)**		**% of iMg** **from tMg**		**% of iMg** **from cMg**	
		**Mean**	**SD**	**Mean**	**SD**	**Mean**	**SD**	**Mean**	**SD**	**Mean**	**SD**
Body mass index (Kg/m^2^)											
Normal(18.5-24.9)	112	2.32	0.49	0.95	0.32	1.10	0.83	39.59	9.91	48.81	5.97
Overweight/Obese(≥25)	144	2.34	0.48	0.95	0.33	1.14	0.91	39.86	8.87	49.44	9.67
Systolic blood pressure (mm Hg)											
<140	215	2.33	0.47	0.95	0.31	1.08	0.85	40.12	9.11	48.65	7.71
>140	41	2.35	0.54	0.91	0.37	1.32	0.96	37.81	10.08	51.99	10.48
Diastolic blood pressure (mm Hg)											
<90	229	2.33	0.49	0.95	0.33	1.10	0.87	39.91	9.22	48.80	7.65
>90	27	2.35	0.42	0.93	0.32	1.30	0.95	38.40	9.95	52.38	12.12
Blood lead levels (µg/dL)											
<40	207	2.33	0.48	0.96	0.32	1.13	0.88	39.91	9.46	49.18	9.07
>40	49	2.24	0.50	0.89	0.32	1.10	0.85	39.07	8.59	49.17	3.30
Smoking											
Yes	57	2.33	0.49	0.98	0.34	0.91	0.82	41.21	8.78	46.91	9.78
No	199	2.33	0.48	0.94	0.32	1.18	0.88	39.33	9.41	49.83	7.71
Alcohol consumption											
Yes	134	2.33	0.46	0.97	0.30	1.03	0.80	40.80	10.30	49.05	9.90
No	122	2.33	0.51	0.93	0.35	1.24	0.90	38.60	8.16	49.33	6.06

The serum magnesium fractions such as tMg, iMg cMg and % of iMg from tMg were negatively associated with BLLs in the study group. In the control group, the levels of tMg and iMg was negatively associated with BLLs and the levels of cMg, %of iMg from tMg and % of iMg from cMg was positively associated with BLLs (Table 5).

**Table 5:** Spearman correlation coefficient(r) between blood lead levels and serum magnesium fractions in study and control group

**Variables**	**Study group**	**Control group**
Blood lead levels (µg/dL)	1.000	1.000
Serum total magnesium(mg/dL)	-0.036	-0.026
Serum ionized magnesium(mg/dL)	-0.066	-0.022
Serum corrected magnesium (mg/dL)	-0.084	0.025
% of Ionized Mg from total Mg	-0.091	0.153
% of Ionized Mg from corrected Mg	0.091	0.110

## Discussion

The present study assessed the effects of Pb- exposure on serum magnesium fractions in workers exposed to Pb from Pb- battery plant. The measurement of BLLs was used as body burden of Pb-exposure. The Pb- exposure among study and control groups was assessed by using the OSHA regulation. 72.8% study group workers had BLLs < 40 µg/dL and 27.2% workers had BLLs >40µg/dL. In case of control group, 100% of workers had BLLs < 40 µg/dL. The magnesium deficiency was associated with hypertension, arrhythmia, arterial calcification, atherosclerosis, heart failure and an increased risk for thrombosis. The magnesium deficiency is a principal driver of cardiovascular disease and public health crisis^21^.

A study, related to short-term Pb-exposure showed an increased serum tMg^10^. The chronic Pb-exposure and Pb-poisoning cases showed decreased serum tMg concentration^11-15^. During the present study, we noticed decreased serum tMg concentration in the study group as compared to control. The condition of hypomagnesium was dependent on protein concentration in sample^4^. The incidence of hypomagnesaemia overestimates through the measurement of serum tMg when hypoalbuminemia is reported^5^. The determination of serum tMg does not reflect the biologically active Mg^2+^ fraction. A reduced level of serum tMg was related to lifestyle factors such as obesity^19^, smoking^22^, alcoholism^34^, high blood pressure^35^ and diabetes^22^. The present study has chosen the serum magnesium fractions such as tMg, iMg, cMg, % of iMg from tMg and % of iMg from cMg to assess the effect of Pb-exposure and lifestyle factors among workers from Pb- battery plant. The parameters of iMg, cMg, % of iMg from tMg and % of iMg from cMg were obtained from serum tMg and serum albumin concentration. During the present study, we noted decreased levels of serum magnesium fractions (tMg, cMg, iMg, % of iMg from tMg and % of iMg from cMg) in the study group as compared to controls. Significantly altered abnormal frequencies of serum iMg and % of iMg from tMg levels were noted in the study group. The serum magnesium fractions such as tMg, iMg, cMg and % of iMg from tMg were negatively associated with BLLs in the study group. In control group, the levels of tMg and iMg was negatively associated with BLLs and the levels of cMg, % of iMg from tMg and % of iMg from cMg was positively associated with BLLs in controls.

Some studies reported decreased serum tMg concentration in smokers^21,36^. Niemela et al^37^ reported decreased serum iMg in smokers. During the present study, we assessed the influence of smoking habit on serum Mg fractions and found significantly decreased levels of cMg and % of iMg from cMg. Among smokers, the levels of serum tMg,iMg and % of iMg from tMg were not altered. Smoking does not affect the serum mineral levels including magnesium^38^. Significantly reduced serum tMg and iMg concentration was reported in chronic alcoholics^6^. In the present study, alcohol consumption was noted significantly decreased in cMg levels. The alcohol consumption pattern among these subjects does not influence serum magnesium fraction because these subjects were not chronic alcoholics.

## Conclusion

The Pb-exposure and lifestyle factors such as smoking and alcohol consumption were associated with alteration of serum magnesium fractions.BMI, DBP, and SBP did not influence serum Mg fractions.

## Conflict of interest statement

The authors declare that there is no conflict of interests.

## Funding

The study was financially supported by National Institute of Occupational Health, Indian Council of Medical Research, Meghani Nagar, and Ahmadabad.

## Highlights

- The present study assessed the serum magnesium fractions: corrected Mg (cMg), ionized Mg (iMg), % of iMg from tMg and % of iMg from cMg in study and controls with contemplation of lifestyle factors.
- The levels of serum tMg, cMg, iMg, % of iMg from tMg and % of iMg from cMg were decreased in study group as compared to controls.
- The serum tMg, iMg, cMg and % of iMg from tMg were negatively associated with blood lead levels.
- Smoking habit was significantly associated with decreased serum cMg and % of iMg from cMg.
- Biologically active forms of serum iMg and % of iMg from tMg were significantly decreased in Pb- exposure.

## References

[1] Naithani M, Bharadwaj J, Darbari A (2014). Magnesium: The fifth electrolyte. J Med NutrNutraceut.

[2] Workinger JL, Doyle RP, Bortz J (2018). Challenges in the Diagnosis of Magnesium Status. Nutrients.

[3] Zaidenberg G, Mimouni FB, Dollberg S (2004). Effect of bicarbonate on neonatal serum ionized magnesium in vitro. Magnesium Res.

[4] Kulpmann WR, Gerlach M (1996). Relationship between ionized and total magnesium in serum. Scand J Clin Lab Invest Suppl.

[5] Saha H, Harmoinen A, Karvonen AL, Mustonen J, Pasternack A (1998). Serum ionized versus total magnesium in patients with intestinal or liver disease. Clin Chem Lab Med.

[6] Hristova EN, Rehak NN, Cecco S, Ruddel M, Herion D, Eckardt M, Linnoila M, Elin RJ (1997). Serum ionized magnesium in chronic alcoholism: is it really decreased?. Clin Chem.

[7] Mauskop A, Altura BM (1998). Role of magnesium in the pathogenesis and treatment of migraines. Clin Neurosci.

[8] Jeremias A, Bertschat FL, Ising H, Jeremias E (2000). Possible correlation between decrease of ionized magnesium and calcium in blood to patient outcome after acute myocardial infarction. J Clin Exp Cardiolog.

[9] Porta S, Epple A, Leitner G, Frise E, Liebmann P, Vogel WH (1994). Impact of stress and triiodothyronine on plasma magnesium fractions. Life Sci.

[10] Dobrakowski M, Boron M, Birkner E, Kasperczyk A, Chwalinska E, Lisowska G (2017). The effect of a short-term exposure to lead on the levels of essential metal ions, selected proteins related to them, and oxidative stress parameters in humans. Oxid Med Cell Longev.

[11] Anetor JI, Ajose OA, Adebiyi JA, Akingbola TS, Iyanda AA, Ebesunu MO, Babalola OO, Aadeniyi FA (2007). Decreased thiamine and magnesium levels in the potentiation of the neurotoxicity of lead in occupational lead exposure. Biol Trace Elem Res.

[12] Chiba M, Shinohara A, Matsushita K, Watanabe H, Inaba Y (1996). Indices of lead-exposure in blood and urine of lead-exposed workers and concentrations of major and trace elements and activities of SOD, GSH-Px and catalase in their blood. Tohoku J Exp Med.

[13] Kristal-Boneh E, Froom P, Yerushalmi N, Harari G, RibakJ RibakJ (1998). Calcitropic hormones and occupational lead exposure. Am JEpidemiol.

[14] Hermann J, Lonchamp P, Duc M (1985). Magnesium metabolism in chronic lead poisoning: influence of chelating treatment. Magnesium.

[15] Ramaswamy P, Kurre M, Muller D, Dargan P, Gevers E, Allgrove J (2015). Hypomagnesaemia due to lead poisoning in the context of a heterozygous CLDN-16 mutation [abstract]. Endocrine Abstracts.

[16] Todorovic T, Vujanovic D, Dozic I, Petkovic-Curcin A (2008). Calcium and magnesium content in hard tissues of rats under condition of sub chronic lead intoxication. Magnesium Res.

[17] Moniuszko-Jakoniuk J, Jurczuk M, Galazyn-Sidorczuk M, Brzoska MM (2003). Lead turnover and changes in the body status of chosen micro-and macroelements in rats exposed to lead and ethanol. PolJEnviron Stud.

[18] Cunha AR, Umbelino B, Correia ML, Neves MF (2012). Magnesium and vascular changes in hypertension. Int J Hypertens.

[19] Shamnani G, Rukadikar CA, Gupta V, Singh S, Tiwari S, Bhartiy SS (2018). Serum magnesium in relation with obesity. Natl J Physio Pharm Pharmacol.

[20] Aziz HS, Blamoun AI, Shubair MK, Ismail MM, DeBari VA, Khan MA (2005). Serum magnesium levels and acute exacerbation of chronic obstructive pulmonary disease: a retrospective study. Annals Clin Lab Sci.

[21] DiNicolantonio JJ, O’Keefe JH, Wilson W (2018). Subclinical magnesium deficiency: a principal driver of cardiovascular disease and a public health crisis. Open Heart,.

[22] Wahid A, Verma GC, Meena CP, Pathan AR (2017). Study of serum magnesium level in patients with type 2 diabetes mellitus and it’s correlation with glycosylated hemoglobin and diabetic complications. Int J Adv Med.

[23] Larsson SC, Burgess S, Michaëlsson K (2018). Serum magnesium levels and risk of coronary artery disease: Mendelian randomisation study. BMC Med.

[24] Sendhav SS, Kakaiya A, Chatterjee B (2017). Evaluation of Serum Magnesium Level along with Lipid Profile in a Gujarati Population diagnosed with Diabetes Mellitus. Indian J Med Biochem.

[25] Mudawi SAA, Ahmed SM, Al-Abd BAH (2013). Assessment of the levels of serum iron and magnesium in sudanese cigarette smokers. IOSR J Pharm.

[26] Shah NC, Liu JP, Iqbal J, Hussain M, Jiang XC, Li Z (2011). Mg deficiency results in modulation of serum lipids, glutathione, and NO synthase isozyme activation in cardiovascular tissues: relevance to de novo synthesis of ceramide, serum Mg and atherogenesis. Int J ClinExp Med.

[27] Altura BM, Shah NC, Shah G, Zhang A, Li W, Zheng T, Perez-Albela JL, Altura BT (2012). Short-term magnesium deficiency upregulatesceramide synthase in cardiovascular tissues and cells: cross-talk among cytokines, Mg^2+^, NF-κB, and de novo ceramide. Am J Physiol Heart Circ Physiol.

[28] Altura BM (1994). Introduction: importance of Mg in physiology and medicine and the need for ion selective electrodes. Scand J Clin Lab Invest Suppl.

[29] Barman T, Kalahasthi R, Rajmohan HR (2014). Effects of lead exposure on the status of platelet indices in workers involved in a lead-acid battery manufacturing plant. J Exp Sci Environ Epidemiol.

[30] National Research Council. Potential Health Risks to DOD Firing-Range Personnel from Recurrent Lead Exposure. Washington, DC: The National Academies Press. 2013. 24901199

[31] (1998). Clinical guidelines on the identification, evaluation, and treatment of overweight and obesity in adults: executive summary. Am J Clin Nutr.

[32] Chobanian AV, Bakris GL, Black HR, Cushman WC, Green LA, Izzo Jr JL (2003). The seventh report of the joint national committee on prevention, detection, evaluation, and treatment of high blood pressure: the JNC 7 report. JAMA.

[33] Doumas BT, Watson WA, Biggs HG (1971). Albumin standards and the measurement of serum albumin with bromcresol green. Clin Chim Acta.

[34] Poikolainen K, Alho H. Magnesium treatment in alcoholics: a randomized clinical trial. Subst Abuse Treat Prev Policy. 2008: 3: 1. 10.1186/1747-597X-3-1 PMC2265283 18218147

[35] Guerrero-Romero F, Rodríguez-Morán M, Hernández-Ronquillo G, Gómez-Díaz R, Pizano-Zarate ML, Wacher NH (2016). Network of childhood obesity of the Mexican Social Security Institute Low serum magnesium levels and its association with high blood pressure in children. J Pediatr.

[36] Khand F, Shaikh SS, Ata MA, Shaikh SS (2015). Evaluation of the effect of smoking on complete blood counts, serum C-reactive protein and magnesium levels in healthy adult male smokers. J Pak Med Assoc.

[37] Niemela JE, Cecco SA, Rehak NN, Elin RJ (1997). The effect of smoking on the serum ionized magnesium concentraiton is method-dependent. Arch Pathol Lab Med.

[38] Meral I, Akdemir FN (2015). Serum mineral status of long-term cigarette smokers. ToxicolInd Health.

